# Chlorogenic Acid-Induced Gut Microbiota Improves Metabolic Endotoxemia

**DOI:** 10.3389/fendo.2021.762691

**Published:** 2021-12-16

**Authors:** Xiaolin Ye, Yang Liu, Jiajin Hu, Yanyan Gao, Yanan Ma, Deliang Wen

**Affiliations:** ^1^ Department of Social Medicine, Institute of health sciences, China Medical University, Shenyang, China; ^2^ Department of Gastroenterology, Beijing Children’s Hospital, Capital Medical University, National Center for Children’s Health, Beijing, China

**Keywords:** ob esity, gut microbiota, chlorogenic acid, lipopolysaccharide, metabolic endotoxemia, insulin resistance

## Abstract

**Background:**

Coffee can regulate glucose homeostasis but the underlying mechanism is unclear. This study investigated the preventive and therapeutic effects of chlorogenic acid (CGA), a polyphenol that is found in coffee, on obesity and obesity-related metabolic endotoxemia.

**Method:**

Male 4-week-old C57BL/6 mice were fed either normal chow or a high-fat diet or 20 weeks and half the mice in each group were gavaged with CGA. Oral glucose tolerance tests (OGTTs) and insulin tolerance tests (ITTs) were performed. Markers of inflammation and intestinal barrier function were assayed. The composition of the gut microbiota was analyzed by 16S rRNA high-throughput pyrosequencing. The role of CGA-altered microbiota in metabolic endotoxemia was verified by fecal microbiota transplantation.

**Results:**

CGA protected against HFD-induced weight gain, decreased the relative weight of subcutaneous and visceral adipose, improved intestinal barrier integrity, and prevented glucose metabolic disorders and endotoxemia (*P <*0.05). CGA significantly changed the composition of the gut microbiota and increased the abundance of short chain fatty acid (SCFA)-producers (e.g., *Dubosiella*, *Romboutsia*, *Mucispirillum*, and *Faecalibaculum*) and *Akkermansia*, which can protect the intestinal barrier. In addition, mice with the CGA-altered microbiota had decreased body weight and fat content and inhibited metabolic endotoxemia.

**Conclusion:**

CGA-induced changes in the gut microbiota played an important role in the inhibition of metabolic endotoxemia in HFD-fed mice.

## Introduction

The worldwide incidence of obesity has risen in recent decades together with the rapid economic development of societies and changes in lifestyle and dietary habits. About 2 billion adults worldwide are estimated to be overweight or obese, and the proportion of overweight and obese children is also on the rise (1). Obesity results from a long-term positive energy balance, an ongoing increased food intake and decreased energy expenditure, and the influence of genetic and environmental factors. Long-term obesity leads to a series of metabolic diseases, such as type 2 diabetes, cardiovascular disease, nonalcoholic fatty liver disease, hypertension, malignant tumors, and others has become a global public health threat (2).

Obesity is characterized by chronic low-grade inflammation. In animal models, high-fat diets (HFDs) eventually lead to gut microbiota imbalance, an increase of intestinal permeability, and entry of lipopolysaccharide (LPS) into the blood, inducing the release of proinflammatory factors, such as TNF-α, IL-1β and monocyte chemotactic protein (MCP)-1 (3, 4). The increase of LPS and release of proinflammatory cytokines results in metabolic endotoxemia, which promotes the development of metabolic syndrome, including impaired insulin signaling and chronic low-grade inflammation. Inhibiting the occurrence of chronic low-grade inflammation would be significant in the management of obesity (5, 6).

Because obesity is largely influenced by lifestyle, dietary habits play an important role in its prevention and treatment. Chlorogenic acid (CGA) is a widely distributed polyphenol with a high content in coffee, fruits, and vegetables. It has been reported to have multiple health benefits including antioxidant and anti-inflammatory properties (7–9). Recent human and animal studies have shown that CGA can reduce body weight and decrease serum total cholesterol (TC), total triglyceride (TG) levels (10, 11). A twice-daily dietary supplement based on CGA improved blood glucose, insulin sensitivity, and other metabolic parameters (TC, TG, and visceral adipose tissue, etc.) in overweight patients (12).

However, as most of those studies have been observational, the underlying mechanisms of those effects have not been determined. To address that issue, we investigated the effects of CGA on obesity, gut microbiota, and metabolic endotoxemia. The purpose of this study was to verify the effectiveness of CGA in the treatment of metabolic syndrome.

## Methods

### Animals

Procedures involving animals followed the guidelines of the Institutional Animal Care and Use Committee of China Medical University Affiliated Shengjing Hospital and were approved by same (approval no. 2020PS034K). Four-week-old male C57Bl/6 mice were purchased from Beijing HFK Bioscience Co. Mice were kept in a specific pathogen-free hospital facility with a 12-h dark/light cycle, a feeding temperature maintained at 18°C to 19°C, and a humidity maintained at 40% to 70%. Mice were fed normal chow and water for 1 week before being randomly divided into four groups. The groups included normal chow with 150 mg/kg CGA dissolved in water and administered daily (NCGA), normal chow with the water vehicle by garage (NFD), an HFD with 150 mg/kg CGA administered daily by gavage (HCGA), and HFD with water by gavage. The general condition, food, and water intake of the mice were observed every day. The mice were weighed every week. Food intake was measured as previously described (13). The mice were euthanized after 20 weeks of treatment.

### Fecal Microbiota Transplantation

Ten-week-old male C57Bl/6 mice were acclimated to a normal chow for 1 week and then randomly divided into four groups that were transplanted with HFD-group microbiota (HFD-R), or HCGA-group microbiota (HCGA-R). Mice in the microbiota transplantation group were given an antibiotic cocktail and then recolonized with donor microbiota as previously reported (13).

### Oral Glucose Tolerance Test (OGTT)

Mice were fasted for 12 h before taking a blood sample from the tail vein for measurement of blood glucose concentration at time zero, after which 1.5 g/kg of a 0.4 g/ml glucose solution was rapidly gavaged. The blood glucose concentration was measured and recorded with a glucometer at 30, 60, 90 and 120 min after injection, a blood sugar versus time curve was calculated and the mice returned to a normal diet.

### Insulin Tolerance Test (ITT)

The mice were fasted for 6 hours before taking a blood sample from the tail vein followed by intraperitoneal injection of 0.75 µ/kg of a 0.2 µ/mL saline solution of insulin. The blood glucose concentration was measured and recorded at 15, 30, 60 and 120 min, a blood sugar versus time curve was calculated, and the mice were returned to a normal diet.

### Biochemical Analysis

Serum levels of tumor necrosis factor (TNF)-α, interleukin (IL)-1β, monocyte chemoattractant protein (MCP)- 1, and insulin were determined with commercial enzyme-linked immunosorbent assay kits (Boster Biological Technology, Wuhan, China) read at OD 450 nm. Concentrations were determined by comparison to standard curves. Plasma lipopolysaccharide (LPS) concentrations were determined using a chromogenic Limulus amebocyte lysate endotoxin assay kit (ToxinSensor, GenScript) as previously described (5).

### Intestinal Epithelial Barrier Permeability *In Vivo*

After 12 h fasting, the mice were given 600 mg/kg fluorescein Isothiocyanate-dextran (80 mg/mL) by gavage. Blood samples were collected before and 2 h after gavage. Plasma fluorescence was measured at an excitation wavelength of 490 nm and an emission wavelength of 520 nm as previously described (13).

### Real-Time Quantitative Polymerase Chain Reaction (qPCR)

Trizol (Invitrogen) was used to extract total RNA and PrimeScript RT reagent kits (TaKaRa, Mountain View, CA) were used to reverse transcribe the RNA samples. The qPCR assays were performed with SYBR Premix Ex Kit (TaKaRa) and a Bio-Rad IQ5 system as previously published using the primers described in **Supplemental Table I** (14).

### Western Blot Assays

Total protein was extracted with radioimmunoprecipitation assay and phenylmethane sulfonyl fluoride lysate buffers. The protein concentrations were determined with a bicinchoninic acid protein assay kit. The western blotting procedures were performed as previously described (14). The blots were incubated with primary antibodies (**Supplemental Table II**) at 4°C overnight. The membranes were incubated with secondary antibodies for 2 h. The blots were visualized using enhanced chemiluminescence substrate kits (Thermo Fisher Scientific, Rockford, IL, USA), and the results were read and analyzed with Image J software (https://imagej.net/Welcome).

### Histological Analysis

Tissues were fixed with 4% paraformaldehyde solution, paraffin-embedded, and cut into 4 μm serial sections. Immunofluorescence was carried out as previously reported (15), intestinal tissue sections were incubated with Claudin-1 (Abcam), diluted at 1:200 at 4°C for 12 hours, and then incubated with a secondary antibody for 1 h. Images were captured by a laser scanning fluorescence microscope (TCS SP5, Leica, Germany) at 200× magnification.

### Gut Microbiota Analysis

Total genome DNA was extracted from six samples from each study group using sodium dodecyl sulfate and cetyltrimethylammonium bromide. The 16S ribosomal (r)RNA genes of distinct regions (16S V3-V4) were amplified using specific barcoded primers. Polymerase chain reaction (PCR) assays were carried out with 15 µL of Phusion High-Fidelity PCR Master Mix (New England Biolabs), 0.2 µM of forward and reverse primers, and about 10 ng of template DNA. Thermal cycling consisted of initial denaturation at 98°C for 1 min, followed by 30 cycles of denaturation at 98°C for 10 s, annealing at 50°C for 30 s, and elongation at 72°C for 30 s and 72°C for 5 min. Sequencing libraries were generated using TruSeq DNA PCR-Free Sample Preparation Kits (Illumina, USA) following the manufacturer’s recommendations; index codes were added. The library quality was assessed with a Qubit@2.0 Fluorometer (Thermo Scientific) and Agilent Bioanalyzer 2100 system. Finally, the library was sequenced on an Illumina NovaSeq platform and 250 bp paired-end reads were generated. Microbiome sequencing data were analyzed with QIIME, a plugin-based microbiome analysis platform. Briefly, raw sequencing reads with exact barcode matches were assigned to respective samples and identified as valid sequences. Paired-end reads were merged using FLASH, Quality filtering on the raw tags was performed under specific filtering conditions to obtain high-quality clean tags according to the QIIME quality-control process. After chimera detection, the remaining high-quality sequences were clustered into operational taxonomic units (OTUs) at 97% sequence identity by UCLUST. Alpha (Chao1 and Shannon) and beta diversity metrics (weighted UniFrac) and principal component analysis (PCA) were calculated with QIIME software. LEfSe analysis was carried out for comparisons among intergroup samples. An alpha significance level of 0.05 and an effect size threshold of 4 were used for all biomarkers (16).

### Statistical Analysis

Data were analyzed with SPSS 21.0 (IBM Corp., Armonk, NY, USA) Continuous variables were reported as means ± standard deviation. Student’s *t*-tests were used to compare between-group differences. Differences among three or more groups were compared by analysis of variance with Bonferroni’s *post-hoc* test. Bar plots were generated with GraphPad Prism 8.0 (GraphPad Software, San Diego, USA). *P*-values < 0.05 indicated statistical significance.

## Results

### CGA Inhibits HFD-Induced Body Weight Gain

**Figure 1:**
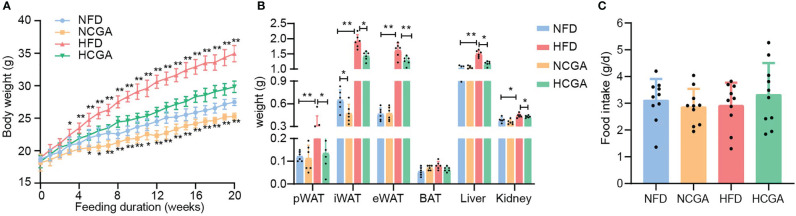
CGA inhibits HFD-induced body weight gain and adipose tissue accumulation. **(A)** Body weight, **(B)** weight of inguinal white adipose tissue (iWAT), perirenal (p)WAT and epididymal (e)WAT, brown adipose tissue, liver, and kidneys, **(C)** food intake, Data are means ± standard deviation. ^*^ *P* < 0.05, ^**^ *P* < 0.01.

After 20 weeks of treatment, the mean body weight of CGA-treated mice was significantly lower than that of the controls (**Figure 1A**). In HFD-fed mice, the reduced weight gain of those given CGA was mainly attributable to a significant reduction in overall fat mass that included both subcutaneous and visceral adipose tissue (**Figure 1B**). Differences in the food intake of the four groups were not significant, indicating that the weight loss induced by CGA was not caused by a reduction in energy intake (**Figure 1C**). These results indicated that CGA treatment inhibited body weight gain and adipose tissue accumulation in HFD-fed mice without limiting food intake.

### CGA Improves Glucose Homeostasis and Insulin Sensitivity in HFD-Fed Mice

**Figure 2:**
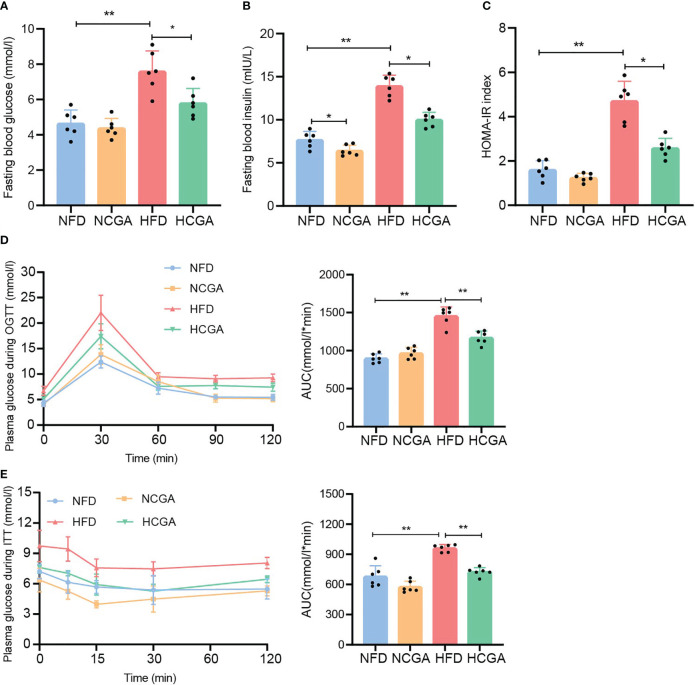
CGA improves glucose homeostasis in HFD-fed mice. **(A)** fasting glucose, **(B)** fasting insulin, **(C)** HOMA-IR index calculated using fasting glucose and insulin, **(D)** oral glucose tolerance test (OGTT), and **(E)** insulin tolerance test (ITT). Data are means ± standard deviation, ^*^ *P* < 0.05, ^**^ *P* < 0.01.

It is well known that insulin resistance is a key component of metabolic syndrome along with T2DM and obesity. To study the effect of CGA on insulin resistance induced in mice by different dietary patterns, fasting glucose, fasting insulin, homeostasis model assessment of insulin resistance (HOMA-IR) index, OGTT, and ITT were measured in mice fed normal chow or the HFD with or without CGA. The results showed that fasting blood glucose, fasting insulin concentrations and the HOMA-IR index in the HCGA group were significantly lower than those in the HFD group, and insulin sensitivity and glucose tolerance were higher than those in the HFD group. Differences between the NCGA and NFD groups were not significant (**Figures 2A–E**). These results showed that CGA treatment improved glucose homeostasis and reduced IR in mice fed with the HFD.

### CGA Inhibits Low-Grade Inflammation

**Figure 3:**
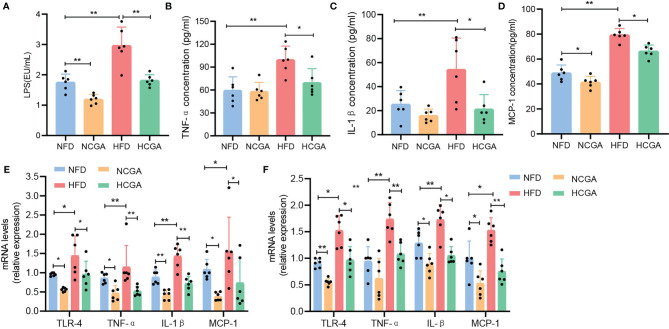
CGA inhibited low-grade inflammation as shown by **(A)** circulating LPS levels. **(B–D)** Serum TNF-α, Il-1β, and MCP-1 cytokine levels **(E, F)** proinflammatory cytokine expression in liver and epididymal fat. Data are means ± standard deviation. ^*^ *P* < 0.05, ^**^ *P* < 0.01.

Obesity is associated with low-grade chronic inflammation, and that may contribute to many associated complications, such as insulin resistance (IR) and subsequent type 2 diabetes (17, 18). LPS is a normal component of the outer cell wall of Gram-negative bacteria, and long-term exposure to LPS results in the development of low-grade inflammation (19, 20). In this study, we compared the plasma LPS levels in four groups of mice. plasma LPS was significantly increased in the HFD group compared with the NFD group, and CGA-treated mice had lower plasma LPS levels than their controls (**Figure 3A**). After LPS enters the bloodstream, it binds to Toll-like receptor 4 on the surface of immune cells to form a complex that promotes the occurrence and development of an inflammatory response (21). To investigate whether CGA improves IR and obesity by regulating the TLR-4 pathway, we assayed TLR-4 expression in liver and epididymal fat. The results indicated that the expression of TLR4 in liver and epididymal adipose tissue from CGA-treated mice was significantly lower than that in the corresponding control group (**Figures 3E, F**). To further investigate whether CGA inhibited LPS-induced low-grade inflammation, we assayed the expression of the proinflammatory mediators TNF-α, IL-1β, and MCP-1in serum, liver, and epididymal adipose tissue from obese mice with IR. The results showed that TNF-α, IL-1β, and MCP-1 levels in serum, liver, and epididymal adipose tissue in the HFD group were significantly higher than those in the NFD group, with lower expression levels in CGA-treated mice than in their controls (**Figures 3B–F**). These results suggested that CGA could inhibit low-grade chronic inflammation in mice.

### CGA Improves the Intestinal Barrier

**Figure 4:**
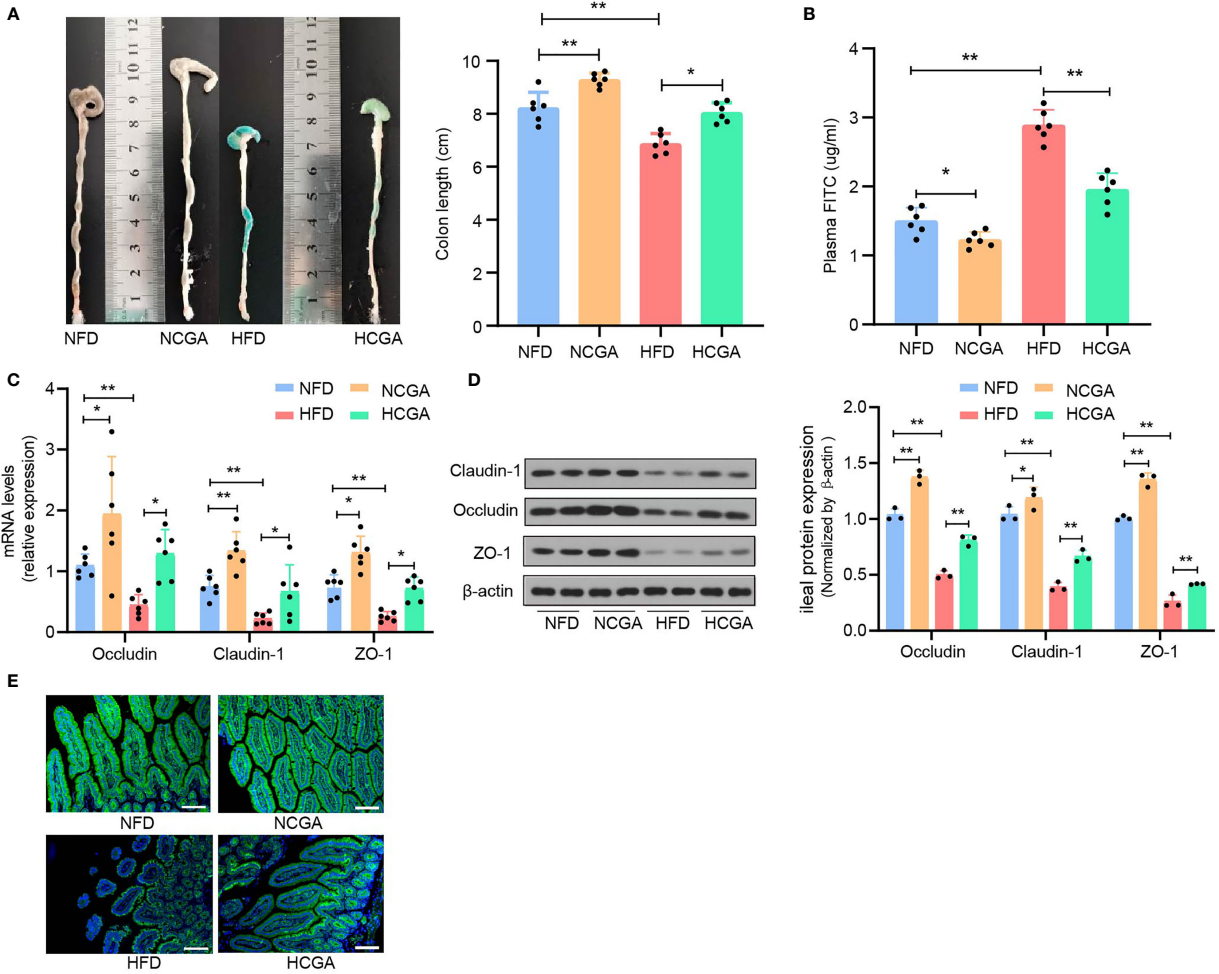
CGA treatment improves the intestinal barrier. **(A)** Representative photographs show the length of colon, **(B)** plasma FITC-Dextran levels, **(C)** claudin-1, occludin, and ZO-1 mRNA expression in the ileum, **(D)** claudin-1, occludin, and ZO-1 protein expression in the ileum, **(E)** representative images of claudin-1.^*^ *P <* 0.05, ^**^ *P <* 0.01.

Previous studies have confirmed that impaired intestinal mucosal barrier function and increased intestinal permeability in people with obesity-related related metabolic syndrome is responsible for entry of LPS into the blood circulation (22, 23). Intestinal epithelial tight junctions are a major component of the intestinal mechanical barrier, maintaining intestinal permeability and integrity. The main proteins involved in the formation of tight junctions are occludin, claudins, and junctional adhesion molecules. The three transmembrane proteins and peripheral cytoplasmic zonula occludens (ZO) proteins form tight junctions. Injury to the intestinal mechanical barrier leads to increased intestinal permeability that may allow translocation of intestinal bacterial, entry of LPS into the bloodstream, and the occurrence of enteric endotoxemia (24). The effects of CGA on intestinal permeability and expression of tight-junction proteins were investigated. We found that the colons of mice in the HFD group were significantly shorter than those in the NFD group, and that the colons of CGA-treated mice were longer than those in their control group (**Figure 4A**). Assay of intestinal mucosal permeability found that the plasma fluorescein isothiocyanate (FITC)-dextran level was significantly higher in HFD group than it was in the NFD group, The plasma FITC-Dextran level was significantly lower in the CGA treatment group than that in the corresponding control group. The results indicated that CGA improved intestinal mucosal permeability (**Figure 4B**). In addition, mRNA and protein expression of the tight-junction proteins (ZO-1, occludin, and claudin-1) in the ileum were increased in CGA-treated mice compared with the control mice (**Figures 4C, D**), Similar trends were observed in claudin-1 immunofluorescence observed by confocal microscopy (**Figure 4E**). These results suggested that CGA could inhibit damage to the intestinal mucosal barrier in mice fed an HFD and reduced the amount of LPS entering the blood circulation.

### Effects of CGA Treatment on the Diversity of Gut Microbiota

**Figure 5:**
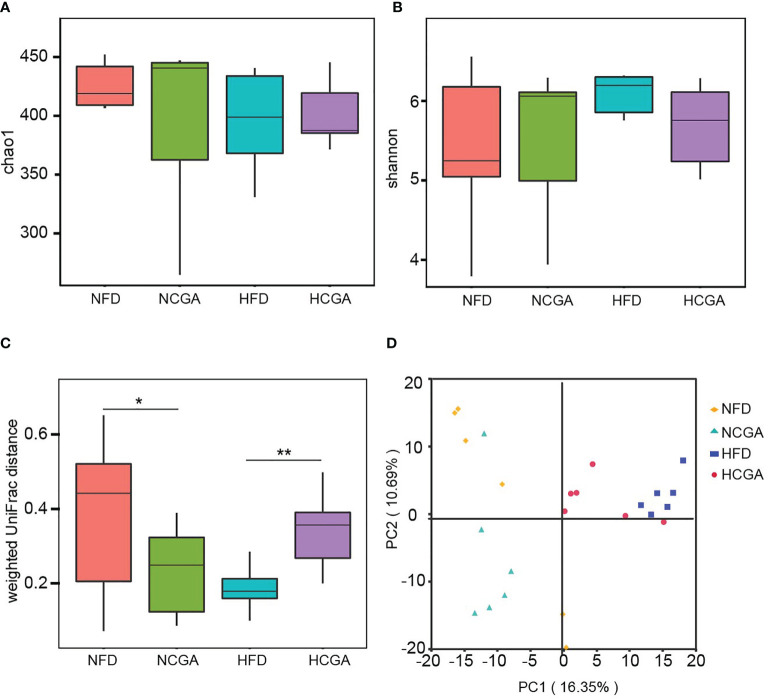
CGA treatment reshaped the gut microbiota of the mice as shown by **(A)** Chao index α-diversity, **(B)** Shannon index α-diversity, **(C)** weighted UniFrac β-diversity, and **(D)** principal component analysis (PCA) of the gut microbiota metagenomes. ^*^ *P* < 0.05, ^**^ *P* < 0.01.

The effects of CGA treatment included an analysis of the changes in gut microbiota composition. Richness estimates obtained from the observed number of species by extrapolation using Chao1 and Shannon indices showed that CGA had no significant effect on the richness of the gut microbiota (**Figures 5A, B**). β-Diversity calculated with weighted UniFrac algorithms indicated that the CGA treatment groups had significant structural differences in the first spatial dimension compared with their controls (**Figure 5C**). PCA revealed distinct clustering of intestinal microbiota communities within each group (**Figure 5D**).

### Effects of CGA Treatment on the Gut Microbiota Composition

**Figure 6:**
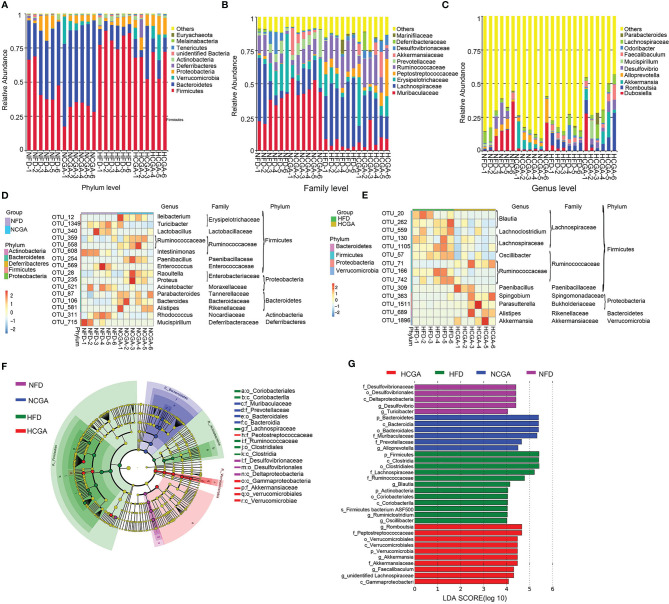
Taxonomic differences in the gut microbiota among the study groups. Six mice were evaluated in each group. **(A–C)** Relative abundance distribution at phylum, family and genus levels. **(D, E)** The heatmap indicates the abundance of 30 OTUs that were significantly changed by CGA. Under-represented units are blue; over-represented units are red. **(F, G)** LEfSe analysis showed phylogenetic differences induced by CGA treatment. Only taxa meeting a linear discriminant analysis significance threshold > 4 are shown.

At the phylum level, *Firmicutes* and *Actinobacteria* were significantly increased, and *Bacteroidetes, Verrucomicrobia*, and *Proteobacteria* were significantly decreased in the HFD group compared with the NFD group. CGA treatment decreased *Firmicutes* and increased *Bacteroidetes* and *Verrucomicrobia* compared with their controls. The results indicated that CGA intervention resulted in a phylum-level shift in the gut microbiota of mice fed the HFD, toward the population seen in the mice fed a normal diet. Effects of CGA on the gut microbiota at the family and genus level are given below (**Figure 6A**).

At the family level, Muribaculaceae, Erysipelotrichaceae, Prevotellaceae, Deferribacteraceae, and Desulfovibrionaceae were decreased, and Lachnospiraceae, Peptostreptococcaceae and Ruminococcaceae were increased in the HFD group compared with the NFD group. The NCGA group had a higher abundance of Muribaculaceae, Prevotellaceae, and Akkermansiaceae and a lower abundance of Lachnospiraceae, Erysipelotrichaceae, Peptostreptococcaceae, Desulfovibrionaceae, and Deferri-bacteraceae than the NFD group. The HCGA group had a higher abundance of Akkermansiaceae, Muribaculaceae, Erysipelotrichaceae, Peptostreptococcaceae, Desulfovibrionaceae, and Deferribacteraceae and a lower abundance of Lachnospiraceae, Ruminococcaceae, and Marinifilaceae than the HFD group (**Figure 6B**).

At the genus level *Dubosiella, Akkermansia, Alloprevotella*, and *Desulfovibrio* were decreased, and *Romboutsia, Mucispirillum, Odoribacter*, and *Faecalibaculum* were increased in the HFD group, compared with the NFD group. The NCGA group had a higher abundance of *Akkermansia* and *Alloprevotella* and a lower abundance of *Dubosiella, Romboutsia, Desulfovibrio*, and *Faecalibaculum* than the NFD group. The HCGA group had a higher abundance of *Dubosiella, Romboutsia, Akkermansia, Desulfovibrio, Mucispirillum*, and *Faecalibaculum*, and a lower abundance of *Odoribacter* than the HFD group (**Figure 6C**).

Analysis of the changes in OTUs showed that compared with the NFD group, Ileibacterium, Paenibacillus, Raoultella, Proteus, Parabacteroides, Bacteroides, and Alistipes were increased in NCGA group, and Turicibacter, Lactobacillus, Intestinimonas, Enterococcus, Acinetobacter, Rhodococcus, and Mucispirillum were decreased in NCGA group. Compared with the HFD group, Paenibacillus, Spingobium, Parasutterella, Alistipes, and Akkermansia were increased in HCGA group, and Blautia, Lachnoclostridium, Oscillibacter, Lachnospiraceae, and Ruminococcaceae were decreased in HCGA group (**Figures 6D, E**).

Linear discriminant analysis effect size (LEfSe) revealed the phylotypes responsible for the differences among the study groups. *Desulfovibrio* and *Turicibacter* were most abundant in the NFD group, *Alloprevotella* was most abundant in the NCGA group, *Oscillibacter*, *Ruminiclostridium*, and *Blautia* were most abundant in the HFD group, and *Romboutsia*, *Akkermansia*, and *Faecalibaculum* were most abundant in the HCGA group (**Figures 6F, G**).

### Effects of CGA-Derived Gut Microbiota on Obesity

**Figure 7:**
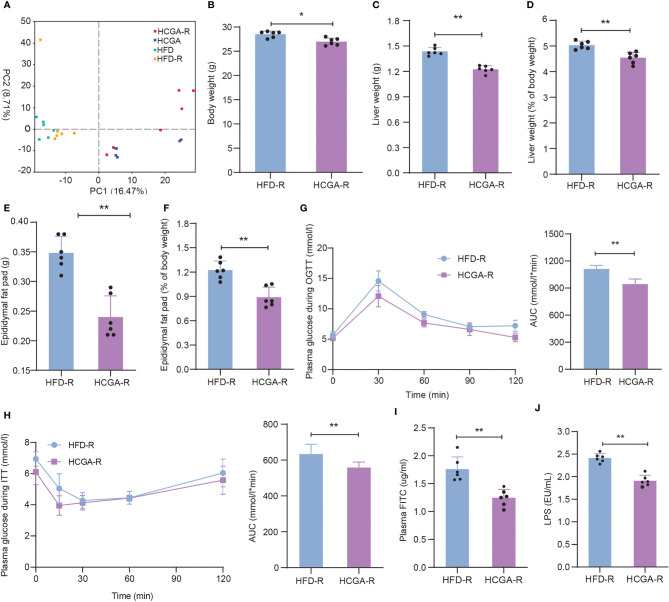
Effects of CGA-derived microbiota on obesity and metabolic endotoxemia. **(A)** PCA of the gut microbiota metagenomes, **(B)** Body weight, **(C)** liver weight, **(D)** liver percentage of whole body weight, **(E)** epididymal fat pad weight, **(F)** epididymal fat pad percentage of whole body weight, **(G)** OGTT, **(H)** ITT. **(I)** Plasma FITC level. **(J)** Plasma LPS level. Data are means ± standard deviation. *P < 0.05, **P < 0.01.

The effects of CGA-derived microbiota on obesity and metabolic syndrome were evaluated by fecal microbiota transplantation. After microbiome depletion treatment, C57BL/6 mice were transplanted with cecal and colon contents obtained from HFD, and HCGA mice. PCA of the gut microbiota composition of the recolonized HFD (HFD-R), and HCGA (HCGA-R) mice was similar to that of the original donor (**Figure 7A**). The relative abundance distribution of gut microbiota in the study four groups at the phylum, family and genus levels are shown in **Supplementary Figure 1**. As shown in **Figures 7B–F**, the HCGA-R group had a lower body weight, liver weight, liver percentage of whole body weight, epididymal fat pad weight, and epididymal fat pad weight percentage of whole body weight compared with HFD-R group. The results indicate that the changes in the microbiota caused by CGA resulted in reduced body weight and body fat percentage in the HFD-fed mice. We further compared glucose metabolism in the two groups, finding that compared with the HFD-R group, mice in the HCGA-R group had better glucose tolerance and insulin sensitivity (**Figures 7G–H**) and that CGA-altered microbiota were associated with significantly improved insulin sensitivity and glucose tolerance in the C57Bl/6 mice. The study confirmed that CGA inhibited the increase of intestinal mucosal permeability, reduced plasma LPS levels, and ultimately inhibited chronic low-grade inflammation. Next, we further clarified the role of CGA-altered gut microbiota in intestinal mucosal permeability. As shown in **Figures 7I, J**, the plasma FITC-dextran and LPS levels were significantly lower in the HCGA-R group than in the HFD-R group. The results suggest that the CGA-derived microbiota improved intestinal permeability. These data demonstrate that the gut microbiota is a primary mediator of improvements in obesity and metabolic endotoxemia.

## Discussion

CGA is widely distributed in plants and is one of the main polyphenols in the human diet. It has been widely studied for its health-promoting effects (7). In this study, the role of gut microbiota in the protective effect of CGA on obesity and metabolic endotoxemia was identified in mice. We found that CGA may inhibit metabolic endotoxemia by regulating gut microbiota, thus improving obesity and IR.

In this study, we observed that CGA significantly inhibited increases in body weight and body fat content induced in HFD-fed mice without affecting the total food intake. Ma et al. reported that CGA significantly blocked the development of diet-induced obesity but did not affect body weight in obese mice (25). Mice were given 150 mg/kg CGA for up to 20 weeks, a larger dose and longer duration than used by Ma et al., and that might be the reason for the difference between our results and theirs. Furthermore, in this study, CGA prevented HFD-induced subcutaneous and visceral adipose weight gain, which is consistent with the findings of previous studies (25, 26). We conclude that the effect of CGA on weight gain mainly resulted from the loss of adipose weight. As visceral fat is strongly related to obesity and its complications, the data suggest that the beneficial effects of CGA may not be limited to weight loss. Additional benefits of CGA are supported by the significant improvements in glucose metabolism, promotion of insulin sensitivity, which are consistent with the findings of Aidilla et al. (27). The study results indicate that CGA treatment inhibited weight gain, reduced fat accumulation, and significantly improved glucose metabolism disorders in obese mice induced by an HFD. The results are consistent with a protective effect of CGA on inhibiting obesity and improving metabolic syndrome, and that the protective effect was not the result of reduced food intake. We plan a further investigation into the mechanism of CGA-improved obesity.

Low-grade inflammation is known to be involved in the pathogenesis of obesity-related metabolic syndrome, insulin secretion defects, and impaired energy homeostasis (28–30). Inflammation of visceral adipose tissue is an important driver of IR (31–33). Mice fed an HFD have higher intestinal permeability and circulating LPS levels than mice fed an NFD, both of which leading to a release of a variety of cytokines and inflammatory mediators (34). On the contrary, lowering the LPS level can reduce the release of proinflammatory factors, downregulate the expression of LPS-related inflammatory proteins, and increase insulin sensitivity (35). In this study, serum LPS concentration, and inflammatory cytokines were higher in the HFD than in the NFD group, which is consistent with previous findings (36). CGA significantly reduced plasma LPS levels, inhibited TLR-4, TNF-α, IL-1β, and MCP-1 expression in liver and epididymal adipose tissue, which further confirmed that CGA reduced low-grade inflammation in the C57Bl/6 mice.

Intestinal mucosal barrier injury is an important cause of metabolic endotoxemia. An intact barrier inhibits the translocation of LPS and bacteria out of the enteric cavity. The structural integrity of tight-junction complexes is key to maintaining the integrity of the intestinal epithelial barrier (37). In this study, after 20 weeks of CGA treatment, intestinal permeability was decreased, ZO-1, claudin-1, and occludin expression in the ileal mucosa were significantly increased. Based on those results, we believe that CGA may inhibit adipose-related low-grade inflammation by improving the intestinal barrier.

The occurrence of chronic low-grade inflammation is closely related to the composition of the gut bacterial community. The imbalance of gut microbiota leads to the increased production and absorption of intestinal endotoxin, causes endotoxemia, promotes the occurrence and development of inflammation, and finally leads to the onset of obesity, IR, T2DM, and other metabolic diseases (6). Compelling evidence shows that there is a link between HFD, body weight regulation, and the gut microbiota (38, 39). Changes in the gut microbiota have been associated with a decrease in the serum LPS level (40). Imbalance in the gut microbiota can lead to an increased uptake of bacterial LPS from the gut into the bloodstream, leading to inflammation, obesity, and IR in obese mice (41). In our study, the gut microbiota of mice fed the HFD had fewer *Bacteroidetes* and more *Firmicutes* bacteria than mice fed the NFD, which are changes that are typical in obesity (42, 43). However, CGA significantly reduced the *Firmicutes/Bacteroidetes* ratio in the gut microbiota of HFD-fed mice, which is consistent with previous studies (26). At the family level, *Bacteroidetes* was reduced in the HFD group and *Lachnospiraceae* and *Ruminococcus* were increased, which is also consistent with previous findings (44). The family *Muribaculaceae* has been shown to inhibit, and *Lachnospiraceae* has been shown to aggravate, obesity. CGA reduced *Lachnospiracea*e and increased the levels of *Muribaculaceae* and *Akkermansiaceae*, which have been negatively correlated with obesity, IR, inflammation, and increased intestinal permeability. In addition, we found that the *Akkermansia*, *Romboutsia*, *Faecalibaculum*, *Dubosiella*, and *Mucispirillum* were significantly increased in mice in the HCGA group compared with those in the HFD group. *Romboutsia, Faecalibaculum, Dubosiella*, and *Mucispirillum* have been shown to produce SCFAs (45, 46). *Akkermansia* belongs to phylum *Verrucomicrobia*, which has been shown to reduce intestinal permeability and maintain intestinal barrier integrity (47). The return to a healthy metabolic state in obese patients after diet restriction may be related to an increase of *Akkermansia* in the gut (48). Gavage of *Akkermansia* in obese mice was reported to inhibit weight gain, IR, and reduce metabolic endotoxemia (49). In addition, *Akkermansia* has significant anti-inflammatory activity. Studies have shown that *Akkermansia* can improve the intestinal mucosal barrier and reduce intestinal inflammation by increasing the intestinal expression of endocannabinoids, proving its important role in maintaining intestinal health and the balance of glucose and lipid metabolism. These results suggest that CGA can improve metabolic endotoxemia by enrichment of SCFA-producing bacteria (e.g., *Dubosiella*, *Romboutsia, Mucispirillum*, and *Faecalibaculum*) and *Akkermansia*, which protects the intestinal barrier.

The effects of CGA-altered microbiota were investigated in C57Bl/6 mice that were transplanted with fecal microbiota from mice in the HFD, and HCGA groups. The transplanted CGA-altered microbiota inhibited body weight gain, decreased adipose tissue content, improved glucose metabolism, and reduced plasma FITC-dextran and LPS level. The results suggest that the CGA-induced changes in gut microbiota are the main reason for the decreased percentage of adipose weight and the inhibited metabolic endotoxemia that occurred. In the future, multi-omics (genomics, metabonomics and transcriptomics) should be used to explore the effects of CGA on gut microbiota structure, intestinal metabolite profile and intestinal mucosal gene expression profile to clarify the effects of CGA on intestinal metabolites and intestinal mucosal core gene expression.

In conclusion, the protective effect of CGA on obesity and related metabolic syndrome was based on its regulation of gut microbiota structure, diversity, and changes in relative abundance at the phylum to genus levels. CGA inhibited systemic low-grade inflammation by increasing the expression of tight-junction proteins in intestinal epithelial cells and inhibiting the translocation of LPS from the enteric cavity into the blood circulation.

## Data Availability Statement

The datasets presented in this study can be found in online repositories. The names of the repository/repositories and accession number(s) can be found below:

NCBI SRA and BioSample: PRJNA715381 and PRJNA766296.

## Ethics Statement

Procedures involving animals followed the guidelines of the Institutional Animal Care and Use Committee of China Medical University Affiliated Shengjing Hospital and were approved by same (approval no. 2020PS034K).

## Author Contributions

Conceptualization, DW. Methodology, XY and YG. Software, XY, JH, and YG. Validation, YL. Formal analysis, XY. Data curation, XY. Writing and preparation of the original draft, XY. Writing, review and editing, DW and YM. Visualization, XY. Supervision, YL. Project administration, DW. Funding acquisition, DW. All authors contributed to the article and approved the submitted version.

## Conflict of Interest

The authors declare that the research was conducted in the absence of any commercial or financial relationships that could be construed as a potential conflict of interest.

## Publisher’s Note

All claims expressed in this article are solely those of the authors and do not necessarily represent those of their affiliated organizations, or those of the publisher, the editors and the reviewers. Any product that may be evaluated in this article, or claim that may be made by its manufacturer, is not guaranteed or endorsed by the publisher.

## Supplementary Material

Supplementary Figure 1 Relative abundance distribution at phylum (a), family (b) and genus (c) levels between HFD-R and HCGA-R mice.
