# Supplementary figures and images for: Chlorogenic Acid-Induced Gut Microbiota Improves Metabolic Endotoxemia
Source: Front Endocrinol (Lausanne). 2021 Dec 16;12:762691. doi: 10.3389/fendo.2021.762691 (PMC8716487; doi:10.3389/fendo.2021.762691)

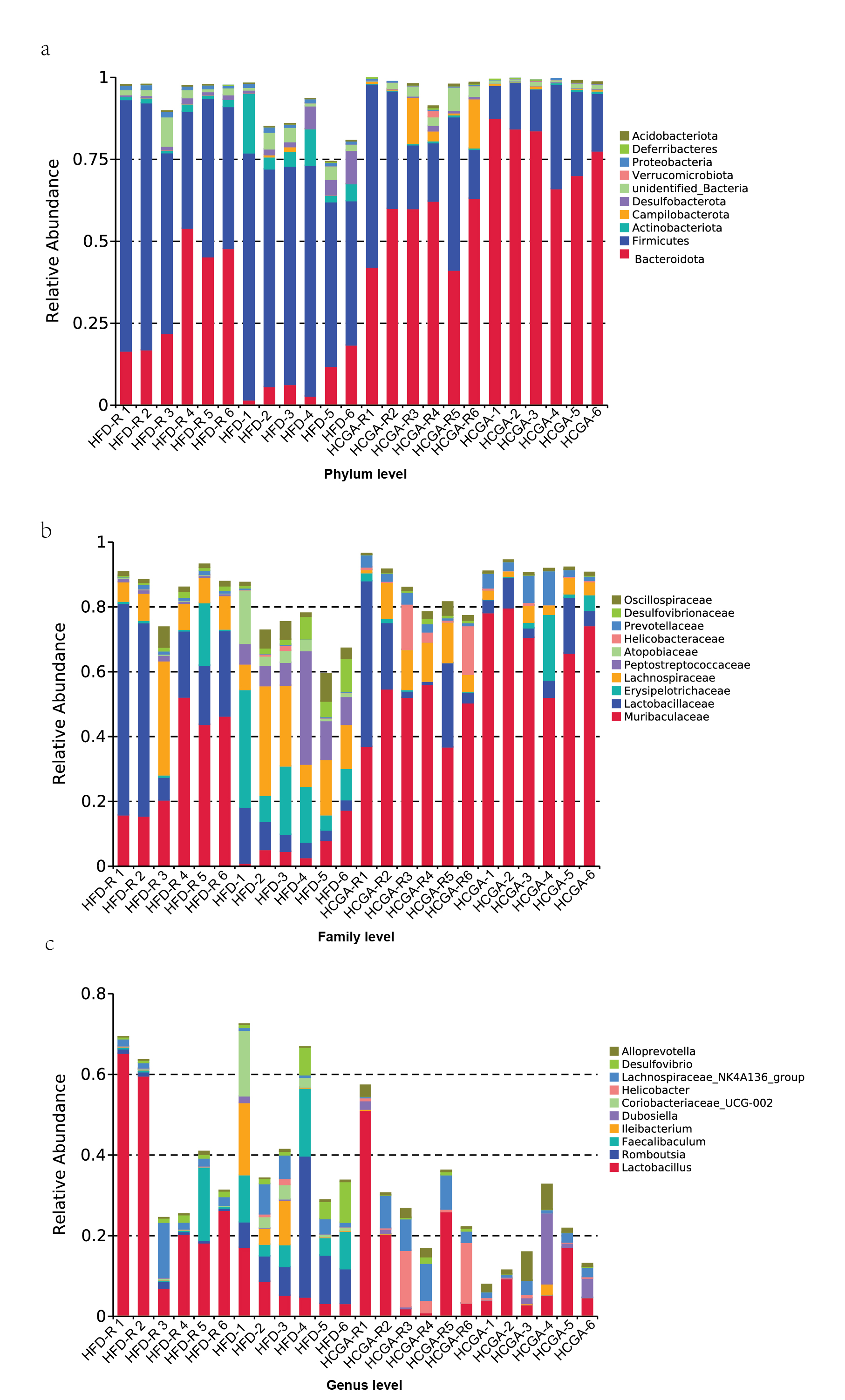

Supplement: Supplementary Figure 1 — Relative abundance distribution at phylum (a), family (b) and genus (c) levels between HFD-R and HCGA-R mice. [file Image_1.jpg]
