# Supplementary material for: Chlorogenic Acid-Induced Gut Microbiota Improves Metabolic Endotoxemia
Source: Front Endocrinol (Lausanne). 2021 Dec 16;12:762691. doi: 10.3389/fendo.2021.762691 (PMC8716487; doi:10.3389/fendo.2021.762691)
Supplement: Supplementary file 2 [file Table_1.docx]

| Primer | sequence 5' to 3' |
| --- | --- |
| TLR4-F | Sense:ATGGCATGGCTTACACCACC |
| TLR4-R | Antisense: GAGGCCAATTTTGTCTCCACA |
| TNFα-F | Sense:AGGAGGAGTCTGCGAAGAAGA |
| TNFα-R | Antisense: GGCAGTGGACCATCTAACTCG |
| IL-1β-F | Sense:ACCTGCTTTCCCCAAAACGAA |
| IL-1β-R | Antisense: TGAGAGAAGTCGCACTGAGTC |
| MCP-1-F | Sense: GGCTCAGCC-AGATGCAGTTAA |
| MCP-1-R | Antisense: CCTACTCATTGGGATCATCTTGCT |
| Occludin -F | Sense: TTGAAAGTCCACCTCCTTACAGA |
| Occludin-R | Antisense: CCGGATAAAAAGAGTACGCTGG |
| Claudin-1-F | Sense:TTGTTTGCAGAGACCCCATC-AC |
| Claudin-1-R | Antisense: GGAGTAAATCTTCCACTGGGGC |
| ZO-1-F | Sense:CAGAACCAAAGCCTGTG-TATG |
| ZO-1-R | Antisense: TTAGGTAGGACACCAT-CAGATGGA |
| GAPDH -F | Sense: GCAAAGTGGAGATTGTTGCCAT |
| GAPDH -R | Antisense: CCTTGACTGTGCCGTTGAATTT |

The primers for PCR.
