# Supplementary material for: Chlorogenic Acid-Induced Gut Microbiota Improves Metabolic Endotoxemia
Source: Front Endocrinol (Lausanne). 2021 Dec 16;12:762691. doi: 10.3389/fendo.2021.762691 (PMC8716487; doi:10.3389/fendo.2021.762691)
Supplement: Supplementary file 3 [file Table_2.docx]

| Primary antibody | Dilution ratio | manufacturer | Art.No. |
| --- | --- | --- | --- |
| Occludin antibody | 1：400 | wanleibio | WL01996 |
| Claudin-1 antibody | 1：500 | wanleibio | WL03073 |
| ZO-1 antibody | 1：500 | wanleibio | WL03419 |
| β-actin | 1:1000 | Cell Signaling Technology | #4970 |

Primary antibodies for WB.
